# Urinary Extracellular Vesicles Biomarkers in CKD: Clinical Laboratory Translation

**DOI:** 10.3390/diagnostics16142181

**Published:** 2026-07-13

**Authors:** Majdi A. Aljohani

**Affiliations:** Department of Medical Laboratory Technology, Faculty of Applied Medical Sciences, University of Tabuk, Tabuk 71491, Saudi Arabia; ma.aljuhani@ut.edu.sa

**Keywords:** CKD, uEVs, liquid biopsy, pre-analytical variability, non-invasive biomarkers, quality management

## Abstract

Globally, chronic kidney disease (CKD) is an increasingly prevalent public health challenge. The current kidney function tests, which include serum creatinine, estimated glomerular filtration rate (eGFR), and proteinuria, are highly useful in clinical practice. Nevertheless, they are characterized by substantial limitations that prevent the early detection of CKD. In contrast, urinary extracellular vesicles (uEVs) may offer an effective alternative for the diagnosis and monitoring of chronic kidney disease if successfully translated. Urinary extracellular vesicles are a wide range of nanosized membrane vesicles that are excreted by cells that line the nephron and urinary tract. These uEVs contain proteins, lipids, and nucleic acids that reflect the pathophysiological state of their cells of origin. This review summarizes the biological evidence for uEV biomarkers in major CKD entities, including diabetic kidney disease, FSGS, IgA nephropathy, ADPKD, and lupus nephritis. From a clinical laboratory perspective, we critically examine pre-analytical variables, analytical factors and validation requirements aligned with ISO 15189 accreditation. We discuss regulatory pathways and the balance between laboratory-developed tests and commercial IVD platforms. Moreover, we conclude that there is an essential need for reference materials, internal quality control, and external quality assessment. Finally, we outline a practical implementation pathway for transitioning uEV assays from research use to routine diagnostics. If successfully translated, uEV-based assays could facilitate earlier detection of CKD, more precise phenotyping, and personalized therapeutic monitoring.

## 1. Introduction

Chronic kidney disease (CKD) is becoming an increasingly significant public health concern globally. The number of individuals affected has more than doubled in the past 30 years [1]. A recent paper reported the global prevalence of CKD at about 14.2%, with 788 million adults aged 20 years and older living with the condition compared with 378 million in 1990. Moreover, CKD accounts for 11.5% of global cardiovascular fatalities in addition to its direct mortality [2]. Nowadays, CKD is recognized in KDIGO guidelines as a major cardiovascular risk condition [3]. Many modifiable risk factors for CKD, including elevated body mass index, high systolic blood pressure, and high fasting plasma glucose, have been identified [2]. Despite this growing burden, kidney care is not universally accessible and affordable, particularly in low-income countries. This inequity presents ethical challenges in regions where renal disease is disproportionately prevalent and resources are scarce [4]. In 2022, the estimated annual direct healthcare costs of diagnosed CKD and kidney transplantation were 372 billion USD across 31 countries. These costs are expected to increase to 406.7 billion USD by 2027, with renal replacement therapy accounting for nearly half of the total costs [5]. Therefore, there is an urgent need to improve the detection and monitoring of CKD, especially in early stages, to reduce disease progression.

The current kidney function tests, including proteinuria, estimated glomerular filtration rate (eGFR), and serum creatinine, are highly useful in clinical practice. However, they have significant limitations that preclude the early detection of CKD [6,7,8]. According to the current KDIGO classification, eGFR and albuminuria are useful in CKD staging but do not fully capture renal pathology [3]. For example, serum creatinine is an imprecise indicator of GFR, as up to 50% of nephron mass may be lost before creatinine levels exceed the normal range [9]. Moreover, its concentration is significantly influenced by non-renal variables, including muscle mass, age, diet, volume status, and analytical variability [10,11]. Similarly, biological and analytical variability in albuminuria and proteinuria has been reported. Furthermore, these markers provide little insight into the specific underlying renal pathology or the nephron segments involved [12,13]. Recent studies have demonstrated that a significant number of patients have CKD despite normal-range proteinuria or minimal albuminuria [12,13]. These kidney biomarkers do not capture the full heterogeneity in disease progression rates or treatment response [14]. Consequently, CKD is frequently identified only after a loss of kidney function that could have been prevented has occurred. Taken together, these limitations of conventional markers suggest that they are inadequate for accurate CKD phenotyping.

In this context, urine is a non-invasive, patient-friendly matrix that directly reflects renal filtration and tubular handling. Moreover, it is readily available in routine clinical laboratories and suitable for long-term repeated sampling. This makes it well-suited for kidney function monitoring and risk verification. Additionally, many biomarkers have been identified, including kidney injury molecule-1 (KIM-1), neutrophil gelatinase-associated lipocalin (NGAL), interleukin-18, monocyte chemoattractant protein-1, and epidermal growth factor [15]. Although these biomarkers have been shown to improve CKD outcomes over creatinine alone in terms of early detection, they have not been incorporated into major CKD guidelines. Nevertheless, these markers have limitations, including susceptibility to non-renal influences such as systemic inflammation [16]. Moreover, these biomarkers show inconsistent diagnostic performance across different clinical settings and limited ability to localize injury at the cellular level [17]. Most of these biomarkers detect a single analyte and therefore capture only one pathway [17]. Taken together, these limitations highlight the need for more accurate and sensitive kidney-specific biomarkers that integrate molecular information. Ideally, these biomarkers should enable early identification with multimodal molecular signals of kidney damage.

**Figure 1:**
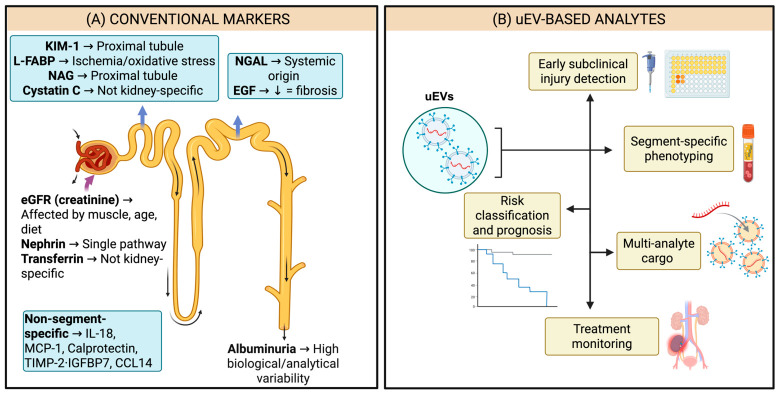
Urinary extracellular vesicles (uEVs) as a next-generation urinary analyte in chronic kidney disease. uEVs are released from a variety of cell types along the nephron and urinary tract, and they contain proteins, lipids, and nucleic acids that are indicative of the pathophysiological state of their cells of origin. The multi-analyte nature of uEVs and their potential to provide segment-specific renal information that complements traditional biomarkers such as serum creatinine and albuminuria are illustrated in the figure.

Given these unaddressed needs, urinary extracellular vesicles (uEVs) offer a promising alternative for diagnosis and monitoring of chronic kidney disease (Figure 1). uEVs are heterogeneous, nanosized membrane vesicles excreted by cells lining the urinary tract [18]. These vesicles contain proteins, lipids and nucleic acids that reflect the physiological and pathophysiological state of their cells of origin [19]. As a result, the analysis of urinary extracellular vesicles offers a kidney-specific approach that provides more molecular information. Several studies have shown that uEVs can detect subclinical injury, distinguish CKD etiologies and correlate with renal function [20,21,22]. Thus, uEVs may significantly enhance the diagnosis and prognosis of chronic kidney disease. Compared with conventional single-analyte urine markers, uEVs simultaneously carry proteins, lipids, and nucleic acids that are involved in multiple signaling pathways [23]. Although uEVs are an advanced study tool, their dynamic molecular composition and diverse origins pose significant methodological challenges [24,25]. Moreover, pre-analytical variability, standardized protocols, and heterogeneity in uEV isolation currently restrict their diagnostic potential [26,27].

In light of this, the main challenge for clinical laboratories is the transition from the discovery of uEV biomarkers to the development of high-throughput, standardized assays. These biomarkers should provide reliable results that can support real-time clinical decision-making. This review aims to provide a practical overview of urinary extracellular vesicles (uEVs) as a novel analyte in chronic kidney disease (CKD), focusing on shifting from fundamental biology to clinical implementation. Specifically, this review synthesizes current knowledge on the biogenesis, renal origin, and pathophysiological relevance of uEVs. This review highlights the key biomarker candidates of uEVs for CKD. Furthermore, we evaluate the pre-analytical and analytical factors that influence the reliability of uEV measurements. Additionally, we identify quality and validation standards for the implementation of uEV assays. Regulatory considerations and practical pathways for integrating uEV testing into clinical laboratory workflows are discussed.

## 2. Biological Evidence for uEVs in CKD

uEVs are nanosized membrane vesicles released from different cells throughout the nephron and urinary tract [28]. They comprise three primary subtypes categorized by their biogenesis, including exosomes, microvesicles and apoptotic bodies. The size of exosomes ranges from 30 to 150 nm, microvesicles from 100 to 1000 nm and apoptotic bodies from 50 to 5000 nm [29]. Notably, MISEV2023 guidelines advise using exosomes only after isolated particles are experimentally confirmed [30]. In CKD biomarker research, most studies have concentrated on the exosomal fraction due to its relative enrichment in kidney-specific molecular components. These uEVs contain proteins, lipids, and nucleic acids that can reflect kidney status. Moreover, they carry a variety of post-translational modifications (PTMs), such as phosphorylation, ubiquitination, and glycosylation [31]. The diagnosis of glomerular and tubular injury may be supported by uEVs when used as a non-invasive tool for CKD [32]. These uEVs may have the ability to differentiate between glomerular, tubular involvement [32], fibrosis progression [33] and subclinical injury [34]. Many studies have demonstrated that uEV protein and miRNA analysis correlate with histologic lesions, albuminuria, eGFR decline, and progression risk [35,36]. However, there are many limitations of this transition, including variability in urine collection, isolation and characterization [37]. Taken together, these findings support the fact that uEVs are clinically promising sources of informative biomarkers in CKD, even though they remain at the exploratory level (Table 1).

### 2.1. Biogenesis and Renal Origin of uEVs

uEVs are small membrane-bound vesicles released into the tubular lumen under both physiological and pathological conditions. They comprise a diverse array of membrane-bound nanoparticles with diameters ranging from 30 to 1000 nm, which include small EVs and larger vesicles [27]. uEVs originate from intraluminal vesicles in multivesicular bodies, which fuse with the plasma membrane to release exosomes [27]. They can also arise from direct budding of the plasma membrane or release of apoptotic bodies because of cellular stress or injury [54]. uEVs collected in the urine are derived from almost all epithelial and stromal cell types along the nephron and urinary tract [28]. This includes proximal tubular, mesangial, podocyte, and glomerular endothelial cells. The percentage release of each segment may vary based on physiological and pathophysiological states [55]. Some studies have hypothesized that blood EVs can be transferred into urine through the renal microvasculature [56]. However, other studies have confirmed that the EV cargo in urine is primarily of kidney origin [57]. The cargo of uEVs is enriched in segment-specific transporters and structural proteins. Some of these are responsible for anchoring uEVs in nephron domains, including ion channels [58], solute carriers [59], and slit-diaphragm components [60]. This provides a spatial and functional understanding of renal biology that may enhance the diagnosis of CKD. Beyond serving as biomarkers, uEVs may work as messengers in the nephron, facilitating communication between different segments [56]. These characteristics establish uEVs as kidney-derived vesicles that both parallel and modulate nephron physiology.

### 2.2. Renal Pathophysiology and Extracellular Vesicles

Since Pisitkun and colleagues first detected and characterized exosomes in human urine, growing evidence has established the correlation between uEVs and CKD [61]. Over the past decade, uEVs have been demonstrated to reflect molecular processes, including podocyte injury, tubular stress, and interstitial inflammation [43,62]. Protein levels and essential transporters in uEVs from different cells across the nephron reflect segment-specific dysfunction [37]. For example, altered expression of tubular transporters indicates proximal or distal tubular stress [37]. In contrast, glomerular damage in diabetic kidney disease is indicated by alterations in slit-diaphragm proteins and podocyte-derived markers [63]. Moreover, they may carry damage-associated molecules, including HMGB1, S100 proteins and regulatory RNAs, that can reflect cellular stress and injury [62]. This links uEV cargo to inflammatory and fibrotic pathways that drive progressive nephron loss [33]. Together, these changes in uEV cargo may diagnose multiple injury pathways, including glomerulosclerosis, inflammation, fibrosis and oxidative stress [36,37,56]. For example, uEV WT1, podocyte-derived proteins, and miRNA profiles correlate with albuminuria and glomerulosclerosis scores [38]. This suggests that uEVs may be involved in the early detection of podocyte stress and the regulation of glomerular injury by RNA in diabetic nephropathy [64]. Moreover, early renal impairment in children with bilateral renal hypoplasia and other congenital kidney and urinary tract abnormalities has been detected by proteomic uEV markers [65]. In addition, others link uEVs to cell-to-cell interaction in which they can transfer profibrotic, pro-inflammatory, or protective signals [33]. Together, these findings establish uEV cargo as a sensitive readout and a potential mediator of kidney injury.

### 2.3. Key uEV Biomarkers in CKD

Over the past decade, several uEV biomarkers have been developed to identify patients at risk of different kidney diseases and correlate with severity and progression risk. These biomarkers include stress score, tubular damage, podocyte-specific proteins, segment-specific transporters, and fibrosis proteins. uEVs contain an extensive range of proteins, lipids, and nucleic acids that reflect renal pathophysiology [33]. The majority of protein biomarkers identified in uEVs are involved in many pathways. For example, α-1-antitrypsin has been reported as a potential protein biomarker for assessing tubular stress and inflammatory activity [66]. Moreover, uEVs have been found to contain podocyte markers, including podocalyxin and WT1, as well as signaling and kinase proteins, including PAK6 and EGFR [67]. Nevertheless, most translational research in CKD has focused on small RNA and uEV proteins [68]. In accordance with this evidence, Table 2 intentionally focuses on uEV protein cargo and small-RNA biomarkers. In small discovery cohorts, many of these uEV-based markers outperform or complement traditional biomarkers, including serum creatinine and albuminuria. This is due to their ability to reflect the intracellular pathophysiological processes before overt functional decline becomes clinically apparent. Furthermore, these markers offer potential advantages over traditional biomarkers, including early detection.

In addition to uEV protein biomarkers in CKD, recent advances in high-throughput sequencing have uncovered a diverse array of dysregulated small non-coding RNAs. For example, a recent paper analyzed 88 participants (20 of them had type 2 diabetes with nephropathy) and concluded that 13 miRNAs were substantially dysregulated, with 10 of them being Piwi-interacting RNAs (piRNAs). It is important to note that miR-151a-3p and miR-182-5p exhibited an opposite expression pattern, as they were reduced in the T2D without nephropathy group. In contrast, it increased in the T2D with nephropathy group, thereby facilitating the differentiation between these two patient groups [69]. Consistent with these findings, a recent investigation identified a unique uEV miRNA profile that was linked to the severity of the disease and histological kidney damage [70]. Two published studies also discussed ADPKD with different methodological approaches. One study focused on exosome-enriched preparations and utilized a discovery–validation approach. This established a correlation between specific uEV signatures and kidney function and progression risk in patients with ADPKD [71]. The second study examined a broader uEV population in a cross-sectional cohort and identified a unique pattern of differentially expressed vesicle payload and potential target pathways. This further emphasizes the biomarker potential of uEVs in ADPKD [72]. From a clinical laboratory perspective, these biomarkers remain in research use only, with no commercial assay approved by regulatory agencies. The cellular origin, pathophysiological association, and clinical utility of representative validated uEV biomarkers in the most common CKD entities are summarized below (Table 2).

**Table 2:** Urinary extracellular vesicle (uEV) biomarkers in chronic kidney disease: research findings and clinical utility.

CKD Entity	uEV Biomarker(s)	Cellular Origin	Pathophysiological Link	Clinical Utility	Prognostic Performance (AUC) *
**DIABETIC KIDNEY DISEASE (DKD)**	**Proteins:** α-1-antitrypsin [66], PAK6 [67] ^a^, EGFR [67], MASP2 [73].	Proximal tubule, glomerular.	Tubular stress, inflammation, complement activation	Early DKD detection before microalbuminuria; limited prospective validation	miR-192: 0.802; miR-194: 0.703; miR-215: 0.757 [74]; PAK6: 0.829; EGFR: 0.797 [67]; miR-136-5p: 0.722 [75].
	**Small RNAs:** miR-145 [76], miR-192-5p [74,77], miR-146a-5p [77], miR-486-5p [77], miR-574-5p [77], miR-194 [74], miR-215 [74], miR-24-3p [78], miR-27b-3p [78], miR-27a-3p [76] ^b^, miR-136-5p [75].				
**FOCAL SEGMENTAL GLOMERULOSCLEROSIS (FSGS)**	**Proteins:** podocalyxin [79], CD63 [79].	Podocyte.	Podocyte injury and foot-process effacement	May distinguish FSGS from other diseases. research-level associations	miR-193a: 0.85 [80].
	**Small RNAs:** miR-193a [80].				
**IGA NEPHROPATHY (IGAN)**	**Proteins:** Vasorin [81], ceruloplasmin [82], aminopeptidase N [81], α1-antitrypsin [81].	Podocyte, glomerular cells.	Glomerular inflammation and mesangial proliferation	May differentiate IgAN from membranous nephropathy and TBMN; research-level associations	miR-451a: 0.817.
	**Small RNAs:** miR-451a [83].				
**AUTOSOMAL DOMINANT POLYCYSTIC KIDNEY DISEASE (ADPKD)**	**Proteins:** MMP-7 [84], Polycystin-1 [85], Periplakin and envoplakin [85], Villin-1 [85].	Proximal tubule, thick ascending limb.	Profibrotic phenotype, cyst expansion, tubular remodeling	May help predict rapid vs. stable disease progression; research-level associations	Not Reported
	**Small RNAs:** miR-192-5p [71] ^c^, miR-194-5p [71], ^d^ miR-320b [72], miR-30a-5p [71] ^d^, miR-1246 [72], miR-146a-5p [71] ^d^, miR-29c [72] ^d^, miR-671-5p [71], miR-30e-5p [71].				
**LUPUS NEPHRITIS**	LINC01127 [86], RUNDC3A [86], glucosylsphingosine [87], PE-NMe [87].	Glomerular and tubular epithelial.	Immune complex-mediated glomerular inflammation and complement activation	May differentiate LN from non-renal SLE, track disease activity; research-level associations	LINC01127: 0.851; RUNDC3A: 0.812 [86]. Glucosylsphingosine: 0.912; PE-NMe: 0.906 [87].
	miR-21 [88], miR-150 [88], miR-29c [88].				

^a^ uEV proteomics (LC-MS/MS); N ≈ 150. ^b^ uEV miRNA sequencing + RT-qPCR; total N ≈ 490. ^c^ uEV miRNA (RT-qPCR), total N ≈ 90. ^d^ Urinary exosome miRNA (small RNA sequencing + RT-qPCR); total N ≈ 82. * AUC values reported in this table derive from discovery-phase studies with limitations, including sample sizes and single centers.

### 2.4. Strengths and Limitations

uEVs are supported as potential biomarkers in CKD by clinical evidence, which includes numerous longitudinal and cross-sectional studies. These studies demonstrate a correlation among histology, albuminuria and eGFR decline and uEV protein and RNA profiles [73,76,79,81,82,84,85,86,88]. Moreover, if successfully implemented in clinical labs, the cost may be lower than invasive procedures such as kidney biopsy. These findings support the clinical relevance of uEVs in nephrology, making them promising tools in clinical laboratories. However, important limitations remain, including a low number of patient samples investigated, few cooperative effects from many centers, and no standardization of isolation and characterization. To our knowledge, no uEV biomarker has yet been validated by trials or incorporated into clinical practice guidelines. Suitable reference materials are still not available, and this can limit how well laboratories can meet certain quality control and standardization requirements. uEVs stand out as attractive options for kidney evaluation when compared to serum creatinine, albuminuria, urine tubular damage indicators, and plasma prognostic biomarkers (Table 3) [33,37,43,89,90]. However, uEV tests lack methodological consistency, regulatory approval, and cost-effective implementation for routine clinical usage as compared to these established markers.

## 3. Pre-Analytical and Analytical Considerations

Pre-analytical variables account for a significant part of laboratory errors and cover the period from the time the physician orders the test to when the sample is received [91]. The pre-analytical phase can determine the validity and clinical usefulness of test results. This phase includes patient preparation, specimen collection, identification, transport, and storage. Numerous studies have demonstrated that even minor discrepancies in urine handling can significantly impact vesicle output, composition, and biomarker assessments in uEVs [24]. On the analytical side, there are many variations in terms of yield and quality based on the isolation methods and normalization used [38]. Analytical platforms introduce additional variability because there are many techniques, including Western blot, flow cytometry, sequencing and mass spectrometry [25]. Therefore, medical laboratory community guidelines, along with stringent internal quality controls and method validation, are needed. This will enable reproducible, comparable, and clinically interpretable uEV biomarkers.

### 3.1. Patient-Related and Urine Collection Variables

In uEVs, the pre-analytical phase is considered a critical source of variability, which includes yield, composition, and interpretability. Many pre-analytical variabilities in uEVs have been studied, including urine storage time and temperature. A recent study showed that storage at −20 °C resulted in the loss of transcripts of EV and protein markers. At −80 °C, the transcriptome was effectively preserved after long-term preservation [43]. Moreover, other authors found that prolonged storage at −20 °C and multiple freeze–thaw cycles reduced EV yield and marker signal intensity [92]. Regarding patient preparation, a recent study investigated the effects of varying fluid intake and dietary conditions on daily urine. The results indicated that uEV counts were substantially impacted by higher fluid intake and variable collection times [93]. This variation appears as physiological differences, including age, sex, and renal function, which lead to significant differences in uEV concentration and cargo [57]. Several groups are currently working on standardized patient preparation and collection to reduce physiological noise and enhance comparability across studies. This standardization is necessary before uEV biomarkers can be translated from discovery studies to clinical laboratory assays.

### 3.2. uEV Isolation Methods Suitable for Clinical Laboratories

The choice of an isolation method is a critical phase in uEV analysis since each technique has specific requirements in terms of equipment, purity, processing time, and yield. These factors directly determine the efficacy of the process for clinical laboratory implementation and its suitability for specific diagnostic applications. Several isolation methods have been reported for uEVs, including differential ultracentrifugation, size exclusion chromatography, ultrafiltration, polymer-based precipitation, and hydrostatic filtration dialysis [27]. These methods differ in purity, processing time, urine volume, and infrastructure demands. Differential ultracentrifugation remains the most widely used reference method for many reasons, including high vesicle recovery [94]. However, its long run time, extensive labor and specialized equipment limit its usefulness in routine diagnostics. One of the most clinically applicable methods among the available alternatives is size exclusion chromatography (SEC), which produces uEVs of high purity with minimal co-isolated protein contamination [94]. Additionally, SEC is designed to be compatible with both proteomic and transcriptomic downstream operations, and it has a rapid turnaround time [95,96]. This makes SEC a suitable candidate for routine testing in clinical laboratories that implement uEVs. However, limitations such as varying recovery of very small EVs and sample dilution should be considered [97]. Current recommendations emphasize that method selection should be driven by reproducibility to ensure valid cross-study comparisons of uEV-derived biomarkers. Table 4 summarizes the practical implications of prevalent uEV isolation techniques in urine.

### 3.3. Minimal Characterization Criteria of uEVs in Clinical Labs

The minimal characterization and analytical measurement of uEVs in clinical investigations should be guided by the general standards for EVs: MISEV 2018 and MISEV 2023 [30]. In addition, urine-specific recommendations issued by the ISEV Urine Task Force should be incorporated to address matrix-specific pre-analytical and analytical issues [43]. At a minimum, preparations should be characterized at particle size and concentration levels, protein levels and nucleic acid or other cargo levels. For quantitative biomarker research, technical performance should be defined in terms of analytical sensitivity, linearity, and precision. This should include an assessment of biological variability and day-to-day repeatability for key uEV cargo. The minimum volume requirements, acceptable turbidity or hematuria levels, and duration from collection to processing should be considered as sample quality criteria [103]. The results should be normalized to urine creatinine or specific gravity and reported with full disclosure of isolation, storage, and measurement conditions. The quality management systems of clinical laboratories should be followed before the implementation process. In this context, uEV tests should be incorporated as defined procedures within a laboratory’s scope of practice. The routine quality system should include the formal validation and documentation of uEV-specific pre-analytical handling, isolation workflows, and analytical performance characteristics.

### 3.4. Normalization Strategies of uEVs in Clinical Labs

Normalization is a critical yet unresolved source of variability in uEV tests, and the suitability of various techniques may vary based on the analytical output, urine content, and kidney function. Creatinine normalization is the most commonly used approach to normalize uEV measurements in many studies [43]. However, it is unreliable in patients with advanced chronic kidney disease, sarcopenia and variable muscle mass due to reduced and variable creatinine excretion [118]. Normalization can be achieved by specific gravity or osmolality measurements, which have advantages in CKD [119]. Many studies have shown that EV content correlates significantly with total urine protein or albumin [120]. Additionally, studies have suggested the use of small RNAs as potential normalizers to account for variations in EV input and isolation efficiency [121]. This is important for downstream mRNA and miRNA assays, as small RNA normalization can help regulate variations in RNA extraction efficiency and reverse transcription [122]. However, it is preferable to combine biomarkers for normalization, as no single systematic normalization strategy is universally suitable.

**Figure 2:**
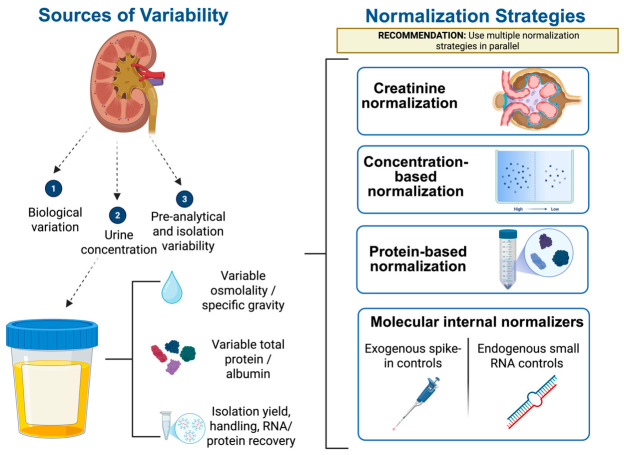
Mechanistic overview of normalization strategies for urinary extracellular vesicle (uEV) biomarkers in CKD. Signal variability is influenced by biological variation, urine concentration, and pre-analytical factors. A more dependable clinical signal can be produced by integrating a variety of normalization methods, such as creatinine, urine concentration (specific gravity/osmolality), total protein, and molecular internal references.

In this context, it is highly recommended to use multiple signals for normalization to get an accurate and specific signal [123]. For example, studies have shown that using creatinine and internal reference transcripts at the same time enhances the accuracy and specificity of the normalization process [124]. Normalization approaches need to be investigated and standardized despite the progress that has been made. Moreover, the impact of various strategies on diagnostic performance metrics, such as sensitivity, specificity, and AUC, must be evaluated. Figure 2 demonstrates the impact of biological variation, urine concentration, and pre-analytical factors on the generation of noise in uEV measurements, in accordance with these principles. It also demonstrates the possible integration of creatinine, concentration, protein, and molecular internal-based normalization strategies to produce more dependable clinical signals.

## 4. Validation and Quality Management of uEVs for Clinical Laboratories

Comprehensive validation is essential to translate uEV tests into clinical laboratory practice. This process should define the intended use and analytical performance specifications (APSs) correlated with CKD-associated clinical decision criteria, as detailed below (Section 4.1) [125]. The within-subject biological variability of an uEV analyte should be considered in analytical performance requirements when possible [126]. The limit of quantitation (LoQ) should be established at or below the lowest clinical decision threshold for accurate early diagnosis of patients. For example, a recent study employed quantitative uEV mRNA measurements to define the utilization of low-abundance uEV transcripts in clinical decision-making for bladder cancer. The analytical measurement range (AMR) should also be verified for different uEV biomarkers in CKD. Moreover, carryover between samples, especially in the context of clinical decisions that are made at or near the threshold, should be monitored [127]. Systematic testing with spike-and-recovery experiments is necessary to address interference from urinary matrix components.

Structured internal quality control for uEVs is required in clinical laboratories, which is achieved using predefined rules and characterized control materials. This control material should include predetermined control rules that are designed to monitor the performance of both the cargo and the particle levels. Furthermore, they should consist of well-characterized vesicle surrogates or defined quantities of urinary extracellular vesicles. Internal quality control should be complemented by external quality assessment systems that are capable of benchmarking performance across platforms and institutions [128]. However, clinical implementation cannot be considered until a number of critical validation limitations are addressed. These include the necessity of disease-specific thresholds, prospective longitudinal studies, independent replication cohorts, and standardized normalization. For example, a recent paper proposed uEV biomarker panels for CKD and childhood kidney diseases that demonstrated disease-associated changes in uEV proteins and RNA cargo [129]. Additionally, direct comparisons should be made with established clinical markers, including eGFR slope, albuminuria (ACR), and kidney biopsy findings. Finally, validation should adhere to the Clinical and Laboratory Standards Institute (CLSI) guidelines, which encompass EP05 for precision, EP06 for linearity, EP07 for interference testing, and EP17 for detection limit determination. Detailed discussions of each of these validation components are provided in Section 4.1, Section 4.2, Section 4.3 and Section 4.4 below.

### 4.1. Intended Use and Analytical Performance Specifications

The clinical purpose of uEV assays is directly determined by their intended use and the required analytical performance specifications (APSs) [130]. uEV assays are intended to detect subclinical renal injury in chronic kidney disease (CKD) applications prior to the occurrence of abnormal conventional markers [131]. Moreover, they may accurately reflect nephron-segment injury, as demonstrated in clinical studies of early tubular and glomerular damage in adults and children. For example, serial uEV protein or RNA signatures can be used to monitor fibrotic progression or treatment response [33]. This refers to the diagnostic accuracy in which patients with a high-normal albuminuria ratio (ACR 10–30 mg/g) are at elevated cardiorenal risk [131]. However, these patients do not receive any therapeutic intervention based on the current CKD criteria (ACR ≥ 30 mg/g). Moreover, the intended use may be to rule out CKD or mentoring therapy to detect true biological change [132]. Disease-specific diagnostic thresholds must be established and validated against clinically meaningful objectives for any intended application, rather than optimizing AUC values in discovery cohorts [133]. These limits must be uniform across a diverse range of patient populations, accounting for comorbidities, sex, ethnicity, and age, which affect the concentration and content of uEVs. User Required Specifications (URSs) are established by the intended use, which must be explicitly defined prior to validation in accordance with international accreditation standards. Analytical performance standards for uEV assays should be based on clinically significant decision thresholds rather than uniform precision targets [125]. Where possible, these standards should also consider within-subject biological variability in the uEV analyte. For example, a uEV assay that is primarily intended as a rule-out test for progressive CKD may be developed to achieve a negative predictive value of over 95% in the target population [133].

In addition to analytical performance specifications, the clinical interpretation of uEV test results requires clearly defined reference intervals and decision thresholds. Reference intervals must be derived from healthy populations and, where appropriate, from disease-specific populations. For example, formal uEV investigations in CKD and childhood kidney disease frequently compare uEV protein and RNA signatures of healthy controls with those of well-defined CKD cohorts to determine disease-associated ranges [134]. Recent data suggests that the index of individuality (IOI) for uEV particle counts measured by NTA is less than 0.6 [126]. This implies that personalized (subject-based) reference intervals may be more suitable for interpreting longitudinal changes in uEV measurements than population-based intervals. Disease-specific decision thresholds must be validated against clinically meaningful endpoints rather than simply optimizing AUC values in discovery cohorts. For example, a uEV marker for early diabetic kidney disease should have a threshold that predicts progression to overt nephropathy, rather than merely correlating with cross-sectional albuminuria [135]. Healthcare professionals should be provided with interpretive guidance that includes the clinical significance of the result, the normalization method used, and the reference interval.

### 4.2. Validation Studies for uEV Assays

Method validation for uEV assays should adhere to general clinical laboratory principles and follow Clinical and Laboratory Standards Institute (CLSI) guidelines, in line with the CLSI-based studies outlined above. In addition, validation should take into account the EV-specific sources of variability in pre-analytical handling and isolation procedures. Precision of particle concentration, protein markers, or miRNA targets should be examined at multiple concentration levels using pooled or artificial samples [126]. Published NTA studies serve as illustrative benchmarks for the analytical precision of EV particle measurements. For example, depending on the sample type and instrument parameters, intra-assay coefficients of variation for particle concentration ranged from 1 to 12%. Day-to-day variation in NTA-based EV measurements reached approximately 25% in that variation study [126,136,137]. Both repeatability and intermediate precision estimates should then be reported [126]. Spike and recovery should be used to determine whether uEV detection is affected by differences in the standard curve diluent and biological sample matrix compared with reference methods [138]. Dilutional linearity experiments on uEV should be performed to demonstrate and verify both proportionality and the absence of matrix effects. Robustness studies should examine the impact of storage conditions on the stability of EVs, isolation protocol parameters, and normalization strategies [139]. This helps ensure that uEV assays are suitable for their intended clinical applications.

### 4.3. Internal Quality Control and External Quality Assessment for uEVs

Internal quality control (IQC) is necessary for the continuous quality assurance of uEV assays after they have been implemented [140]. These systems should be analogous to those used for routine chemistry, hematology, and immuno-assay testing. IQC materials may include stabilized preparations stored at −80 °C, synthetic or recombinant EV surrogates, or aliquoted pools of human urine-derived EVs [24,139,141]. To identify trends, shifts, or increased imprecision in particle counts or cargo measurements, these materials are monitored using Levey–Jennings charts and predefined rules [142]. Both analytical and pre-analytical phases must be subjected to IQC, and control materials should be characterized for commutability and stability over relevant time frames [140]. These are necessary to identify and rectify variability that may have been introduced by changes in isolation reagents, storage conditions, operator techniques, or instrument performance [24,126]. Furthermore, the field requires formalized external quality assessment or proficiency testing schemes for uEV assays. These programs would distribute standardized uEV or EV-mimicking preparations to participating laboratories [43]. This will facilitate the identification of systematic biases between analytical platforms or isolation methods and facilitate inter-laboratory comparisons. At present, there is no formal external quality assessment program that is specifically designed for uEV biomarkers [143]. This is a significant impediment to the widespread implementation and robust clinical validation of these biomarkers.

### 4.4. Reference Materials and Calibrators for uEVs

**Figure 3:**
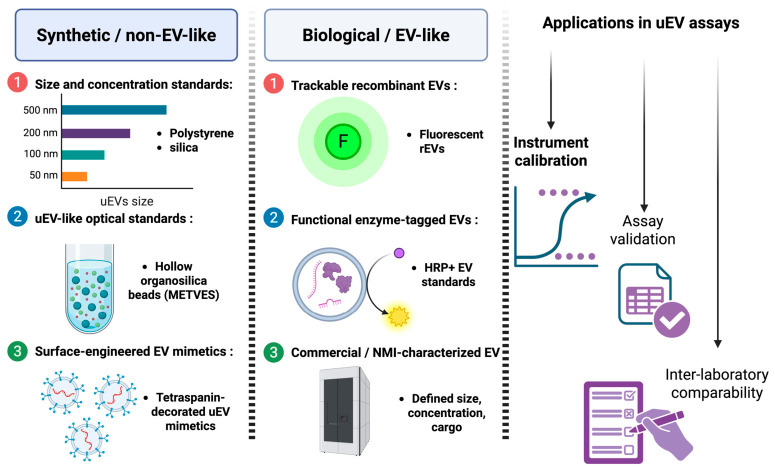
Potential reference materials for uEV assay standardization in chronic kidney disease (CKD). Synthetic nanoparticles, recombinant extracellular vesicles (rEVs), EV-mimetics (tetraspanin-decorated), pooled human urine-derived EVs, and defined protein/RNA standards are all acceptable candidates. These materials are essential for the establishment of traceability, calibration, recovery assessment, and inter-laboratory comparability.

One of the major barriers to the standardization of uEV measurements across clinical laboratories is the lack of reference materials [43]. Several initiatives are underway to establish candidate materials, including synthetic nanoparticles and recombinant vesicles. For example, tetraspanin-decorated EV mimetics have been suggested as adaptable reference materials for standardizing EV phenotyping and quantification measurements [144]. Moreover, a recent study used recombinant extracellular vesicles (rEVs) engineered with fluorescent tags to spike for recovery assessment and data normalization [145]. These materials are intended to serve a variety of functions, including traceability establishment and calibration enabling for different analyzers. This would provide common comparators for multi-center validation studies and define minimum reporting elements for isolation and characterization. Additionally, it is essential to apply rigorous batch-effect control protocols in addition to reference materials in order to identify and rectify inter-batch variability in uEV isolation and analysis [146]. Together, reference materials, calibrators, and standardization frameworks are important components of the field (Figure 3). They are required to convert uEV assays from promising research instruments into robust, interoperable diagnostics. Without these efforts, uEV biomarkers will remain in research settings despite their significant correlation with CKD.

## 5. Regulatory, Accreditation, and Clinical Integration

Transitioning uEV-based CKD biomarkers from research to routine clinical practice is a complicated process. A comprehensive framework is needed to meet regulatory conformance, laboratory accreditation and clinical implementation. In CKD, this framework is particularly important since it may have the ability to detect kidney injury early and monitor disease progression [65]. Moreover, it may help reduce reliance on invasive procedures such as kidney biopsy. However, uEVs have many complex pre-analytical, analytical, and post-analytical phases compared with conventional assays. At present, no regulatory approval has been granted for an uEV-based assay for the diagnosis or monitoring of CKD by major regulators [147]. The regulatory approach for an uEV assay will be dependent upon its intended application and the associated clinical risk, requiring more stringent evidence than that required for lower-risk applications. These require standardization and validation in accordance with established regulatory and quality standards. The validation and implementation of uEV assays should be guided by laboratory accreditation to ISO 15189 [148], which serves as the benchmark for quality competence. Simultaneously, laboratories must determine whether to employ laboratory-developed tests (LDTs) or commercial in vitro diagnostic (IVD) platforms for uEV assays.

### 5.1. Accreditation Considerations for uEVs

uEV assays should be aligned with clinical laboratory accreditation standards in order to transition from study tools to effective diagnostic tests. The quality framework that governs other accredited laboratory tests must be integrated with uEV methods for a smooth transition [149]. Key performance characteristics for uEV assays include imprecision, analytical sensitivity, reportable range, accuracy, traceability, interferences, and stability [141]. Performance specifications of tests for CKD applications should be explicitly defined. These specifications must guarantee that changes in uEV biomarkers correspond to genuine alterations in renal status, rather than analytical variability [150]. For example, at early-injury decision stages, an uEV assay measuring podocyte-derived vesicles carrying nephrin and podocin may be accredited with predefined imprecision and reportable range variables [82]. This ensures that changes in these indicators during follow-up actually represent glomerular injury rather than method variability. To facilitate this transition, the pre-analytical step should be standardized, and SOPs should be documented [151]. Moreover, internal and external quality control should be taken into account for optimal transition.

**Figure 4:**
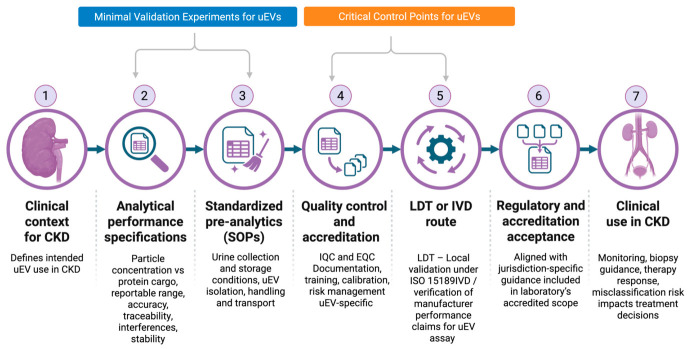
Summary of the implementation pathway from defining the CKD clinical context to routine clinical use of an accredited uEV assay.

In this context, accreditation standards, including ISO 15189, should be followed to ensure the implementation of uEV assays [141]. This requires maintaining comprehensive documentation, staff training and competency records, equipment calibration and maintenance logs, and formal risk management procedures [152]. This is critical when uEV assays are intended to complement or, in selected scenarios, substitute traditional markers such as serum creatinine, albuminuria, or histological assessment [68]. In these situations, misclassification can directly affect clinical decisions about biopsy, treatment escalation, or referral. In addition, reported results must be robust, reproducible, and appropriate for guiding clinical decision-making in chronic kidney disease. Alignment of uEV assays with these accreditations supports regulatory acceptance [153]. The transition from LDTs to fully validated IVD platforms is very significant for the successful implementation of uEV in clinical laboratories. The pathway is summarized in Figure 4, which includes the current clinical needs of CKD, uEV biomarker evidence, ISO 15189 analytical and quality management requirements, and LDT/IVD regulatory guidance. This pathway is intended to define the process by which uEV assays can transition to accredited clinical use for CKD.

### 5.2. Laboratory-Developed Tests Versus IVD Platforms for uEVs

Clinical laboratory tests fall into one of two categories: laboratory-developed tests (LDTs) or in vitro diagnostics (IVDs) [154]. Most clinical laboratory tests are IVDs, which are commercially produced assays. However, most uEV assays in CKD are implemented as laboratory-developed tests (LDTs) [143]. This enables technique optimization and biomarker discovery to meet clinical requirements. In CKD research, many of these LDTs are currently used in observational cohorts or early-phase studies to explore uEV markers associated with diabetic kidney disease, glomerulonephritis, or renal fibrosis [89,155,156]. However, these markers are not yet standardized enough to guide routine clinical decisions. This requires extensive validation, quality monitoring and strong documentation to meet regulatory and accreditation requirements. In contrast, IVD platforms offer standardized reagents, automated workflows, and predefined performance characteristics, which make them easy to adapt in clinical laboratories [143]. However, a very limited number of uEV-based IVD assays have been developed without regulatory approval in CKD [143]. Therefore, the transition from LDTs to fully validated IVD platforms is very significant for the successful implementation of uEVs in clinical laboratories. This remains a critical step for the successful and widespread clinical implementation of uEV diagnostics in CKD. For example, standardized and validated uEV assays are needed for risk stratification, treatment monitoring, and biopsy-sparing diagnostic algorithms. In the current research and early-translation phase, it will be crucial to exercise caution when employing uEV-based LDTs, as well as to implement rigorous quality management and validation procedures.

### 5.3. Integration of uEV Assays into Clinical Laboratories

The implementation of uEV assays into clinical laboratories is currently limited by the few validated studies and automated tests suitable for routine testing. Most studies have focused on the harmonization of pre-analytical workflows, including collection, storage, and isolation [24,26,94,126]. On the other hand, many papers have focused only on a single uEV biomarker for integration into clinical laboratories [143]. Several studies that investigated lupus nephritis, diabetic kidney disease, and cancer have produced uEV panels as non-invasive tools that can be used [33,157]. In CKD, several studies have shown that uEV signatures may distinguish different stages of kidney damage and predict progression or therapeutic response [36,87]. However, these findings are rarely embedded into existing CKD care pathways or laboratory information systems. This highlights the gap between discovery studies of uEVs and clinical laboratory use. It is important to focus on embedding uEV assays into existing lab quality systems. This includes using a reliable source for reporting based on international databases and following accreditation-aligned validation principles (ISEV/MISEV, MIQE, ISO 15189) [29]. It is necessary to take care of the fit of the existing instrumentation, turnaround time requirements, and normalization in routine testing. Ensuring such compatibility will be essential if uEV-based tests are to be incorporated into CKD clinics as routine tools for follow-up, therapy adjustment, and early detection of subclinical renal injury.

## 6. Summary and Future Perspectives

uEVs have been proposed as a clinically promising and biologically attractive tool for the non-invasive diagnosis of chronic kidney disease [46,60]. The content presented throughout this review from different papers and organizations can be summarized in many key statements. Initially, uEVs derive from epithelial and stromal cell types throughout the nephron and urinary tract, possessing segment-specific molecular fingerprints [45,89]. This allows location-specific renal biology profiling that is not accessible through conventional serum or urine biomarkers. Furthermore, the cargo of uEVs, which includes proteins, miRNAs, mRNAs, and lipids, is indicative of the pathophysiological state of their progenitor cells [32,33,34,43,87]. This enables early detection of CKD before the onset of functional decline (Table 2). Moreover, disease-specific uEV biomarkers have been identified in research papers, including diabetic kidney disease, IgA nephropathy, FSGS, lupus nephritis, and ADPKD. In ADPKD, urinary exosomal miRNA signatures establish a mechanistic connection between cyst biology and epithelial injury [71]. However, the results of another uEV study were inconsistent as a result of the variation in cohort composition, methods, and outcome definitions [72]. The potential for these biomarkers to be used in diagnosis is highlighted by their correlation with histological severity and eGFR decline. Applying the National Academy of Medicine (NAM) evidence framework [158], uEVs are still in the early clinical validity stage. Most biomarkers have limited analytical validity under specific laboratory conditions.

From a clinical laboratory perspective, the complete analytical process must be rigorously monitored during the transition of uEV tests toward approved diagnostic tests. In this review, we considered a variety of pre-analytical variables that can introduce substantial variability [26,43,159]. This includes urine collection timing, hydration status, storage temperature, freeze–thaw cycles, and Tamm–Horsfall protein interference. The yield, purity, and compatibility of EVs are significantly influenced by the selection of the isolation method [94,97]. Ultracentrifugation remains the most widely referenced method; however, size exclusion chromatography has become a clinically desirable alternative that provides high purity and reduced protein contamination [97]. The challenge of normalization strategies in the field of uEV biomarker research remains to be resolved. Many studies support the fact that normalization should be used with multiple normalizers, including creatinine and internal molecular reference (Figure 2) [120,123,124]. Overall, these observations highlight the necessity of pre-analytical variable control, the selection of EV isolation methods, and multi-parameter normalization strategies.

Analytical validation of uEV assays should be governed by MISEV2023/ISEV Urine Task Force recommendations and CLSI frameworks. This includes the integration of precision studies, interference testing, dilutional linearity, and clinically based analytical performance specifications. To ensure longitudinal assay performance and cross-laboratory comparability, quality control should be implemented. To our knowledge, no uEV-based assay has received regulatory approval, and most of them may be considered laboratory-developed tests only. One barrier is the low number of certified reference materials or calibrators, which are essential for medical lab implementation (Figure 3). We summarized the ways to integrate uEV assays into laboratory practice with respect to ISO 15189 (Figure 4). Compared with circulating extracellular vesicles and urine cell-free DNA, uEVs distinctly facilitate nephron segment-specific resolution. Nonetheless, their relative therapeutic efficacy has not been determined by direct comparative research.

Beyond these priorities, we need large-scale, prospective, multi-center clinical studies to define disease-specific diagnostic thresholds and establish reference intervals across diverse patient populations. Moreover, the harmonization of pre-analytical protocols is essential for successful validation in clinical laboratories and the development of automated, integrated analytical platforms is essential to meet the turnaround times and throughput requirements of standard clinical laboratory practice. In addition to biomarker development, future investigations should examine the potential of uEVs to dynamically monitor treatment responses and disease progression. Future research must consider whether uEV testing reduces biopsy frequency, prevents hospitalizations, or enables earlier intervention at a lower cost than current standards. Together, these initiatives will be crucial in bridging the divide between experimental research and standard clinical application. This will facilitate the integration of uEVs as a fundamental element of diagnostic nephrology in clinical laboratories.

## Figures and Tables

**Table 1:** Key biological features and clinical relevance of urinary extracellular vesicles (uEVs) in chronic kidney disease (CKD).

uEV Characteristic	Molecular Information Captured	Representative Clinical Associations	Key Biomarkers
*Nephron segment enriched uEV origin*	Cell type-specific proteins and RNAs; glomerular and tubular injury	Differentiation of glomerular versus tubular injury	**Glomerular markers:** Podocalyxin [32,38], WT1 [32], CD133 [39]. **Podocyte markers:** Nephrin [40], Podocalyxin [38], WT1 [32], p16 (CDKN2A) [41], p19 (CDKN2D) [42]. **Proximal tubular:** Megalin [37], Cubilin [43], AQP1 [43], Aminopeptidase N [37], NHE3 [44]. **Loop of Henle**: NKCC2 [37], Uromodulin [45]. **Distal nephron:** SLC12A3 [46], Claudin [33]. **Collecting duct:** AQP2 [47], UT-B [47].
*Pathology and severity of CKD*	Inflammation, fibrosis, and signaling pathways	Correlation with histologic lesions, albuminuria, eGFR decline	**Fibrosis:** TGF-β1 [48], Collagen IV [33], Fibronectin [33]. **Inflammation:** CCL2 [48], HMGB1 [48], TNF-α and IL-6 [49]. **Signaling pathways:** miR-21 and 29 [48], mTOR [36], Wnt/β-catenin [33], NF-κB [50].
*Early clinical and pathway activity*	Pathway level activity beyond conventional markers	Detection of early or preclinical injury changes	**Early tubular injury:** ATF3 [51], TIMP2 [52], IGFBP7 [52]. **Early profibrotic signaling:** miR-21 [48], miR-29 [48].
*Dynamic monitoring and treatment response*	Longitudinal assessment of kidney status and treatment monitoring	Potential for dynamic risk stratification	**Dynamic tubular function:** Sodium Chloride Cotransporter (NCC) [53], ENaC subunits [37], ROMK [47]. **Longitudinal assessment:** SLC27A2 [20], Amnionless (AMN) [20]. **Treatment monitoring:** AQP2 [38], NKCC2 [37].

**Table 3:** Comparative strengths and limitations of biomarkers in chronic kidney disease.

Parameter	Serum Creatinine	Albuminuria	Urinary Tubular Injury Markers	Plasma Prognostic Markers	Kidney Biopsy	uEV Biomarkers
**Detects early injury**	No	Limited	Yes	Yes	Yes	Yes
**Segment-specific**	No	No	Tubular only	No	Yes	Yes
**Biological specificity**	Low	Moderate	Moderate	Moderate	High	Potentially high
**Standardized**	Yes	Yes	Moderate	Moderate	Yes	No
**Cost**	Low	Low	Low	Moderate	Very High	High
**Clinical approval**	Yes	Yes	No	No	Yes	No

Yes = strong evidence/No = not applicable/Limited = some evidence but major gaps/Moderate = partial or conditional.

**Table 4:** Practical considerations for uEV isolation methods in urine.

Parameter	Ultracentrifugation (UC)	Size Exclusion Chromatography (SEC)	Ultrafiltration (UF)	Polymer Precipitation	Immunoaffinity Capture
Yield vs. purity (urine)	High yield, moderate purity [98].	Moderate yield, high purity, may require preconcentration step [99].	Moderate yield and purity (vesicle may be lost on membrane) [100].	High yield, low purity [38].	Low yield, high purity (specific subgroup) [101].
Minimum urine volume	≥9.0 mL urine [102].	<10 mL [103].	10–50 mL [100].	<10 mL [38].	<10 mL [101].
Processing time	Long (2–4 h) [43].	Moderate (30–60 min) [104].	Moderate [100].	Short [105].	Moderate [106].
Uromodulin (THP) interference	Major [107].	Low (most THP elutes in later fractions) [108].	Moderate (can clog membranes) [109].	High (not selectively removed) [108].	Low (specificity) [108].
Hydration/dilution sensitivity	High; dilute urine markedly reduces pellet yield [43].	Moderate (less affected than UC) [99].	High [110].	Low [111].	Low [112].
Scalability (clinical labs)	Poor [113].	Good (semi-automation) [114].	Good (UF devices) [110].	Good (simple) [115].	Limited (high cost) [116].
Downstream compatibility (RNA, protein)	RNA-seq, qPCR, proteomics; contamination possible [43,117].	RNA-seq, qPCR, proteomics, immuno-assays [95,96].	Useful as pre-step before RNA-seq and proteomics [109,110].	Limited (interference) [105].	Excellent for targeted protein assays [106,112].

## Data Availability

No new data were created or analyzed in this study. Data sharing is not applicable to this article.

## Abbreviations

ACR: Albumin-to-Creatinine Ratio; ADPKD: Autosomal Dominant Polycystic Kidney Disease; AMN: Amnionless; AMR: Analytical Measurement Range; APS: Analytical Performance Specifications; AQP1: Aquaporin 1; AQP2: Aquaporin 2; ATF3: Activating Transcription Factor 3; AUC: Area Under the Receiver Operating Characteristic Curve; CCL2: C-C Motif Chemokine Ligand 2; CKD: Chronic Kidney Disease; CLSI: Clinical and Laboratory Standards Institute; DKD: Diabetic Kidney Disease; eGFR: Estimated Glomerular Filtration Rate; EGFR: Epidermal Growth Factor Receptor; ENaC: Epithelial Sodium Channel; EQA: External Quality Assessment; EV: Extracellular Vesicle; FSGS: Focal Segmental Glomerulosclerosis; HMGB1: High Mobility Group Box 1; IGFBP7: Insulin-like Growth Factor-Binding Protein 7; IOI: Index of Individuality; IQC: Internal Quality Control; ISO: International Organization for Standardization; IVD: In Vitro Diagnostic; KDIGO: Kidney Disease: Improving Global Outcomes; KIM-1: Kidney Injury Molecule-1; LC-MS/MS: Liquid Chromatography–Tandem Mass Spectrometry; LDT: Laboratory-Developed Test; LoD: Limit of Detection; LoQ: Limit of Quantification; MIQE: Minimum Information for Publication of Quantitative Real-Time PCR Experiments; miRNA: MicroRNA; MISEV: Minimal Information for Studies of Extracellular Vesicles; MMP-7: Matrix Metalloproteinase-7; mTOR: Mammalian Target of Rapamycin; NCC: Sodium Chloride Cotransporter; NF-κB: Nuclear Factor Kappa B; NGAL: Neutrophil Gelatinase-Associated Lipocalin; NKCC2: Na-K-2Cl Cotransporter; NTA: Nanoparticle Tracking Analysis; PAK6: p21-Activated Kinase 6; piRNA: Piwi-Interacting RNA; QC: Quality Control; RNA: Ribonucleic Acid; ROMK: Renal Outer Medullary Potassium Channel; RT-qPCR: Reverse Transcription Quantitative Polymerase Chain Reaction; SEC: Size Exclusion Chromatography; SLC12A3: Solute Carrier Family 12 Member 3; SLC27A2: Solute Carrier Family 27 Member 2; TGF-β: Transforming Growth Factor Beta; TIMP2: Tissue Inhibitor of Metalloproteinases 2; uEV(s): Urinary Extracellular Vesicle(s); URSs: User Required Specifications; UT-B: Urea Transporter B; WT1: Wilms Tumor Protein 1.
